# 

**Published:** 1872-12

**Authors:** 


### CONTENTS:

ORIGINAL COMMUNICATIONS:

ARTICLE I. The Pathology of Leucocythæmia. By I. N. Danforth, M.D., Chicago .......... 705
ART. II. Exposure and Stretching of the Spinal Nerves. Trans. by Prof. Freer, Chicago .......... 712
ART. III. Items. Collated by J. H. Etheridge, M.D., Chicago .......... 717
ART. IV. Neuralgia. Lecture by Walter Hay, M.D., Chicago .......... 724
ART. V. Ulcer of the Rectum and Anus. By John E. Owens, M.D., Chicago. 733
ART. VI. Therapeutical Notes. By Thos. A. Elder, M.D., Mifflintown, Pa .......... 737
ART. VII. Artificial Placental Respiration. By J. N. Hyde, M.D., Chicago .......... 743

CORRESPONDENCE .......... 744
SELECTIONS .......... 746
EDITORS' BOOK TABLE .......... 757
EDITORIAL .......... 761
LOOT .......... 764

